# Assessment of surfactant use in preterm infants as a marker of neonatal intensive care unit quality

**DOI:** 10.1186/1472-6963-11-22

**Published:** 2011-01-31

**Authors:** Heather C Kaplan, Scott A Lorch, Jennifer Pinto-Martin, Mary Putt, Jeffrey H Silber

**Affiliations:** 1 Perinatal Institute and James M. Anderson Center for Health Systems Excellence, Cincinnati Children's Hospital Medical Center, 3333 Burnet Avenue, Cincinnati, OH, USA; 2 Center for Outcomes Research, The Children's Hospital of Philadelphia, 3535 Market Street, Philadelphia, PA, USA; 3 Department of Pediatrics, Division of Neonatology, The Children's Hospital of Philadelphia, 34th Street and Civic Center Boulevard, Philadelphia, PA, USA; 4 Center for Clinical Epidemiology and Biostatistics, University of Pennsylvania, 423 Guardian Drive, Philadelphia, PA, USA; 5 School of Nursing, University of Pennsylvania, 418 Curie Boulevard, Philadelphia, PA, USA; 6 Departments of Pediatrics, Anesthesiology and Critical Care Medicine, The Children's Hospital of Philadelphia, 34th Street and Civic Center Boulevard, Philadelphia, PA, USA

## Abstract

**Background:**

Proposed neonatal quality measures have included structural measures such as average daily census, and outcome measures such as mortality and rates of complications of prematurity. However, process measures have remained largely unexamined. The objective of this research was to examine variation in surfactant use as a possible process measure of neonatal quality.

**Methods:**

We obtained data on infants 30 to 34 weeks gestation admitted with respiratory distress syndrome (RDS) within 48 hours of birth to 16 hospitals participating in the Pediatric Health Information Systems database from 2001-2006. Models were developed to describe hospital variation in surfactant use and identify patient and hospital predictors of use. Another cohort of all infants admitted within 24 hours of birth was used to obtain adjusted neonatal intensive care unit (NICU) mortality rates. To assess the construct validity of surfactant use as a quality metric, adjusted hospital rates of mortality and surfactant use were compared using Kendall's tau.

**Results:**

Of 3,633 infants, 46% received surfactant. For individual hospitals, the adjusted odds of surfactant use varied from 2.2 times greater to 5.9 times less than the hospital with the median adjusted odds of surfactant use. Increased annual admissions of extremely low birth weight infants to the NICU were associated with greater surfactant use (OR 1.80, 95% CI 1.02-3.19). The correlation between adjusted hospital rates of surfactant use and in-hospital mortality was 0.37 (Kendall's tau p = 0.051).

**Conclusions:**

Though results were encouraging, efforts to examine surfactant use in infants with RDS as a process measure reflecting quality of care revealed significant challenges. Difficulties related to adequate measurement including defining RDS using administrative data, accounting for care received prior to transfer, and adjusting for severity of illness will need to be addressed to improve the utility of this measure.

## Background

Measuring the quality of health care has become a major focus of policymakers, health care purchasers, and physicians. Traditionally, quality of care is measured by one of three dimensions--structure (characteristics of the environment in which care is delivered), process (care itself or what is actually done to a patient), or outcome (the patient's health status). Neonatal quality measures that have been examined include structural measures such as average daily census, and outcome measures such as mortality and rates of complications of prematurity. However, process measures of neonatal quality have remained largely unexamined.

The best processes to use as quality indicators are those linked to improved outcomes through sound scientific evidence [1,2]. Surfactant therapy for neonatal respiratory distress syndrome (RDS) serves as an excellent evidence-based process measure. In both very low birth weight (VLBW, <1500 grams) [3] and larger [4-6] preterm infants, it has been shown to lead to a significant decrease in the risk of mortality and pneumothorax [7,8]. Additionally, position statements from professional organizations support use of this proven therapy in infants of any gestational age with RDS [9-11]. However, while there is documented variation in the evidence-based use of surfactant among VLBW infants [12,13], variation in evidence-based surfactant use among larger preterm infants--the most prevalent group of preterm infants--has not been examined. Additionally, given that a majority of preterm infants are delivered moderately preterm and these patients are not targeted by current quality measures, concerted efforts are needed to identify and validate measures targeting this patient population [14].

The ideal approach to quality measurement incorporates structure, process, and outcome dimensions of quality and utilizes indicators that are valid, reliable, and easy to collect [15]. Rigorous development of potential quality of care measures requires establishing that the measure has (1) face validity--the perception that the measure actually reflects better or worse care; (2) construct validity--evidence that quality as measured by the proposed metric is consistent with quality when measured by other metrics; and, (3) stability over time. It also requires that the proposed metric be adequately measured with available data sources

We aimed to assess whether surfactant use in infants 30 to 34 weeks' gestation with RDS is a valid measure of neonatal intensive care unit quality of care. Face validity was established by identifying support for this process in the medical literature and recommendations from professional organizations [9-11]. The objective of this study was to determine whether significant variation exists in the use of surfactant among infants infants 30 to 34 weeks' gestation across hospitals and to test the construct validity of surfactant use as a quality measure by identifying characteristics associated with higher rates of use and comparing ratings with two measures of quality--volume of admissions [16-18] and in-hospital mortality [16,17,19].

## Methods

The protocol was reviewed by The Children's Hospital of Philadelphia Institutional Review Board (IRB) and was determined to be IRB exempt.

### Data source

Data were obtained from the Pediatric Health Information Systems (PHIS) database developed by the Child Health Corporation of America, a business alliance of freestanding, children's hospitals. PHIS contains data from 40 not-for-profit, tertiary care United States children's hospitals. This database contains demographic information, diagnosis and procedure codes (recorded using the International Classification of Diseases, 9^th ^Revision, Clinical Modification (ICD-9-CM) format) and billed transaction and utilization data which are mapped to standardized Clinical Transaction Classification (CTC) codes. The database also contains all-patient refined diagnosis-related groups (APR-DRGs, version 15) and their associated birth weight groupings, as determined from patients' diagnosis and procedure codes and submitted birth weight (if available) using 3M proprietary software.

### Study population

**Figure 1:**
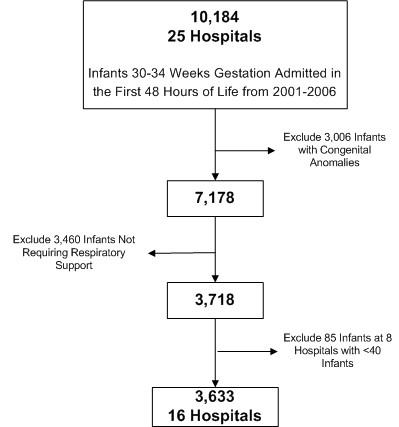
**Inclusion and exclusion criteria applied to identify the study population**. This figure displays how inclusion and exclusion criteria were applied to identify the study population. Prior to obtaining data from PHIS, 11 hospitals that do not submit gestational age information were excluded. From the initial study population of infants 30 to 34 weeks gestation admitted in the first 48 hours of life, exclusion criteria were applied sequentially.

The study cohort consisted of infants born at 30 to 34 weeks gestation with RDS, admitted between January 1, 2001 and March 31, 2006 to a hospital participating in the PHIS database. We defined RDS as requiring respiratory support with nasal continuous positive airway pressure (NCPAP) or mechanical ventilation (MV) in the first 48 hours of life, as determined from billing CTC codes and ICD-9-CM procedure codes. We chose to use a clinical definition of RDS instead of the ICD-9-CM diagnosis code (ICD-9-CM 769) in our primary analysis because assignment of the ICD-9-CM code is done retrospectively and may be biased by whether an infant received surfactant. Additionally, the ICD-9-CM code for RDS has never been specifically validated and it is likely susceptible to coding errors because it requires significant judgment by data abstractors. We excluded infants admitted at >48 hours of life, who would be outside the treatment window for surfactant, and infants with congenital anomalies. We included data only from hospitals that submitted gestational age information. Hospitals that did not submit gestational age were similar to hospitals that did with regard to number of staffed beds, average daily census, and population of the surrounding city. We also included only those hospitals that submitted both billing and clinical data to PHIS. Hospitals with <40 eligible infants in the study period were excluded to minimize unstable estimates of the outcome in small centers. Figure 1 describes how the exclusion and inclusion criteria were applied to obtain the final study population.

### Data quality and missing data

PHIS data are warehoused by a third-party vendor (previously Solucient, Ann Arbor, MI) which loads and processes data submitted by the hospitals. The warehouse partner applies 175 audits to each patient record. Submissions that do not meet the error thresholds are rejected. For example, neonate specific audits include identifying inconsistencies in typical length of stay (LOS) for a given birth weight group (i.e. LOS < 45 days for infant 600-749 grams) and identifying potential misclassifications of preterm and full term infants (i.e. BW < 2500 grams for a full term infant and BW > 2500 grams for a preterm infant). The hospital must correct the errors, resubmit the data, and meet the threshold before the data is included in PHIS.

A majority of the variables had missing data in <5% of observations. Race information was missing for 6% of patients. Among the population used to measure in-hospital mortality rates, 7.5% of patients had a gestational age that was determined to be inaccurate (<22 weeks or >43 weeks) or was missing. Patients with missing data were included in all analyses with variables coded in the regression models as absent (reference), present, and missing. Sensitivity analyses to the missing data were carried out for several aspects of the data analysis using imputed data and the results did not vary substantively from those presented here.

### Outcome of interest

The primary outcome measure was receipt of surfactant within the first 48 hours of life. Surfactant use was identified by presence of an appropriate CTC code for any surfactant (i.e. lung surfactant unspecified, Beractant, synthetic lung surfactant, Calfactant, and Poractant alpha) in the pharmacy billing data. Only surfactant charges with a day of service within the first 48 hours of the infant's life were included.

### Hospital characteristics

#### Hospital identifying characteristics

Hospital factors of interest were the number of annual extremely low birth weight (ELBW) admissions to the NICU, percent Medicaid admissions to the hospital, amount of National Institutes of Health (NIH) research funding, and percent of admissions to the neonatal intensive care unit (NICU) with a principal surgical diagnosis. These variables were calculated for the most recent full year in the database (2005). ELBW admissions were defined using APR-DRG birth weight groupings. Amount of NIH research funding was obtained from the National Association of Children's Hospitals and Related Institutions for the year 2005.

#### Hospital measures used to test construct validity

The measure of volume of admissions used to test construct validity was determined as described above. We also aimed to test construct validity by comparing hospital rates of surfactant use with overall in-hospital mortality rates. This analysis is not intended to make a causal link between surfactant use and mortality at the patient-level but to examine whether rankings based on surfactant use are similar to rankings based on overall in-hospital mortality rates at the hospital-level. Overall NICU rates of mortality were determined using a second cohort of infants from the same hospitals which consisted of 24,421 neonates (any gestational age) admitted to a participating PHIS NICU within 24 hours of birth during the same study period. This second cohort was necessary to provide the correct population for measuring overall in-hospital NICU mortality as an outcome measure of quality. We excluded five patients who lacked a recorded ICD-9-CM procedure or diagnosis code. Physiologic measures of severity of illness were unavailable in the PHIS database and risk adjustment models have not specifically been validated for use with this database. Therefore, we used demographic and clinical variables to control for differences in case-mix across hospitals when calculating adjusted rates of surfactant use and mortality as in previous studies [16,20]. The variables available to control for case-mix in our analyses (a subset of the variables included in previous studies) included gender, presence of any congenital anomaly, race, gestational age, and birth weight <10^th ^percentile for gestational age.

### Statistical analysis

Analyses were carried out using Stata 9 (College Station, TX); hypothesis tests were two-sided and used a Type I error rate of 0.05. Odds ratios (OR) are presented with their 95% confidence intervals (CI). Unless the model included individual hospitals as fixed effects, we used modified Huber-White sandwich estimators to adjust the standard error estimates in the regression models for possible correlations among patients from the same hospital [21].

#### Variation in surfactant use and hospital characteristics associated with use

We built a patient-level logistic regression model for the study population that predicted surfactant use as a function of patient level variables (i.e. gestational age, race, gender, day of life on admission, and mode of ventilation). Indicator variables for specific hospitals were added to the model to estimate odds of surfactant use for each hospital relative to the hospital with the median rate of surfactant use (hospital I), after adjustment for patient characteristics.

#### Comparison with other quality measures--construct validity

We tested the construct validity of surfactant use as a measure of quality by (1) examining the association between surfactant use and hospital characteristics, specifically volume of admissions to the NICU (described in previous section), and (2) comparing adjusted hospital rates of surfactant use with adjusted hospital mortality rates. For the first test, we examined the coefficient of each hospital characteristic after individually adding the hospital-level characteristic to the patient-level model. For the second test of validity, using the population of patients of all gestational ages admitted in the first 24 hours of life, we built a logistic regression model to predict the odds of in-hospital mortality including covariates to adjust for case-mix (described in previous section). Using the logistic regression model to estimate the adjusted expected number of patient deaths (E) for each hospital, we calculated an (O-E)/N statistic for each hospital, where O was the observed number of events (deaths) at a hospital and N was the number of patients at the hospital [22]. We also calculated the (O-E)/N statistics for surfactant use at each hospital using the same methodology. This statistic reflects the excess or reduction in rate of surfactant use (or mortality) of a given hospital compared to an "average" hospital with the same mixture of patient characteristics. A test of whether the observed and expected rates differed was calculated using methods described by Haberman [23]. In addition, as a confirmatory analysis, we used bootstrap techniques [24] to obtain a mean (O-E)/N and 95% confidence interval for each hospital. The association between adjusted hospital mortality rates and adjusted hospital rates of surfactant use was assessed using Kendall's tau probability of concordance (which is calculated by the formula 1+ tau)/2) and the Kendall's tau correlation coefficient [25]. The probability of concordance provides the probability that if a hospital is ranked higher on surfactant use, it will also have a better (lower) adjusted mortality rate.

#### Sensitivity analyses

In order to test whether variation in the odds of surfactant use among hospitals was robust to changes in the definition of RDS, we performed a sensitivity analysis defining RDS with the ICD-9-CM code for respiratory distress syndrome (ICD-9-CM 769) as opposed to the clinically derived definition of requiring ventilatory support (NCPAP or MV) in the first 48 hours of life. Additionally, to examine variation in the patient population specifically targeted by the American Academy of Pediatrics [10] for evidence based surfactant (intubated infants with RDS) and to examine the possible effect of receiving surfactant prior to transfer, we performed a sensitivity analysis examining only the cohort of infants admitted within 24 hours of birth who required mechanical ventilation.

## Results

### Variation in rates of surfactant use

A total of 3,633 infants from 16 hospitals were included in the study population. Mean gestational age was 32.2 ± 1.4 weeks. A majority of patients were male (57.8%) and of white race (70.8%). Overall, 46% of these patients received surfactant. Unadjusted rates of surfactant use among hospitals ranged from 18.9% to 71.4%. Among the 2,961 (81.5%) patients on mechanical ventilation during the first 48 hours of life, 55.6% received surfactant. Seventy-eight percent had an ICD-9-CM diagnosis code for RDS. Other diagnoses included congenital pneumonia (2.3%), meconium aspiration (0.2%), transient tachypnea of the newborn (8.6%), and severe birth asphyxia (0.7%). Of the included hospitals, 15 (94%) were academic centers and 14 (88%) were located in a city with a population >1 million.

Multivariate models indicated that gestational age, race, and gender were significantly associated with surfactant use (Table 1). Requiring mechanical ventilation sometime during the first 48 hours of life, compared to requiring only NCPAP, was most strongly associated with receipt of surfactant (OR 55, 95% CI 36-84). Receipt of surfactant was independent of whether the infant was admitted in the first 24 hours of life (day 0) or in the second 24 hours of life (day 1).

**Table 1:** Predictors of surfactant use in cohort 1 (n = 3,633)*

	Odds Ratio	95% CI	*p*-value
PATIENT-LEVEL PREDICTORS*			

**Gestational age**			

30 weeks (n = 621)	Reference		

31 weeks (n = 620)	0.73	0.57-0.95	0.019

32 weeks (n = 747)	0.68	0.53-0.87	0.002

33 weeks (n = 715)	0.58	0.45-0.75	<0.001

34 weeks (n = 930)	0.69	0.55-0.88	0.003

**Race†**			

Non-white (n = 840)	Reference		

White (n = 2,571)	1.22	1.02-1.47	0.03

**Gender**			

Female (n = 1,535)	Reference		

Male (n = 2,098)	1.34	1.15-1.57	<0.001

**Age at admission**			

Day of life 0 (n = 3,107)	Reference		

Day of life 1 (n = 526)	1.11	0.89-1.38	0.34

**Mode of respiratory support (first 48 hrs)**			

NCPAP (n = 672)	Reference		

Mechanical ventilation (n = 2,961)	54.99	35.86-84.35	<0.001


**HOSPITAL-LEVEL PREDICTORS††**			

**Research involvement**			

Top 10 hospital based on NIH total awards to children's hospitals	1.48	0.98-2.25	0.065

**Surgical volume**			

≥ 29% (median) of admissions to the NICU classified as surgical	1.58	0.79-3.16	0.193

**Payer mix**			

≥ 45% (median) of admissions to the hospital paid by Medicaid	0.80	0.40-1.61	0.532

**Patient volume**			

≥ 45 (median) Annual ELBW Admissions	1.80	1.02-3.19	0.042

NCPAP = nasal continuous positive airway pressure, NIH = National Institutes of Health; NICU = neonatal intensive care unit; ELBW = extremely low birth weight

*Model includes patient-level variables listed in the table plus hospital fixed effects

†Model includes a term for missing race (n = 222)

††Model includes patient-level variables listed in the table plus hospital characteristics added individually

**Figure 2:**
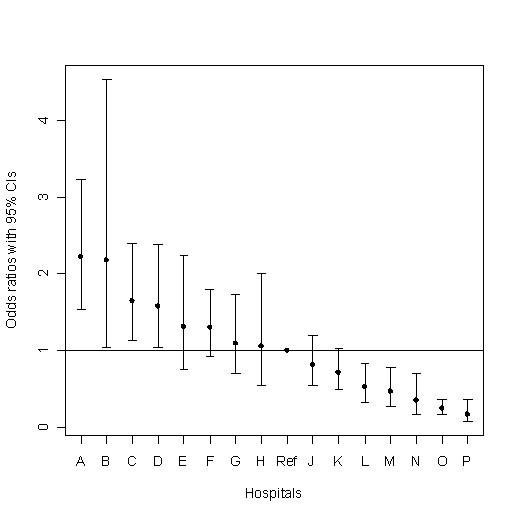
**Adjusted hospital odds of receiving surfactant relative to reference hospital**. This figure displays the hospital's adjusted odds ratio and associated 95% confidence interval (95% CI) of receiving surfactant relative to reference hospital defined as having the median adjusted rate of surfactant. After adjusting for patient characteristics, five hospitals had significantly decreased odds and four hospitals had significantly increased odds of giving surfactant, compared to the median hospital. After Bonferoni correction for multiple comparisons (p ≤ 0.05/16 = 0.003), 4 hospitals remained significantly different than the reference hospital

After adjusting for patient characteristics, five hospitals had significantly decreased odds and four hospitals had significantly increased odds of giving surfactant, compared to the median hospital (Figure 2). Odds ratios varied from as much as 2.2 (95% CI 1.53-3.23) times greater to 5.9 (95% CI 2.72-12.3) times less than the median hospital (p ≤ 0.001).

Among the 2,465 patients admitted within 24 hours of birth who required mechanical ventilation, unadjusted rates of surfactant use ranged from 19.2% to 76.3% across hospitals. Additionally, significant variation in adjusted surfactant use persisted in 7 of the 9 hospitals that were significant in the original analysis (Hospitals A, B, L, M, N, O, and P). Significant hospital variation in surfactant use was also found when the cohort was restricted to infants with an ICD-9-CM diagnosis code for RDS.

### Construct validity

Among hospital-level characteristics, patient volume was significantly associated with variation in receipt of surfactant, while involvement in research approached significance (Table 1). NICU surgical volume and hospital payer mix were not associated with surfactant use.

**Figure 3:**
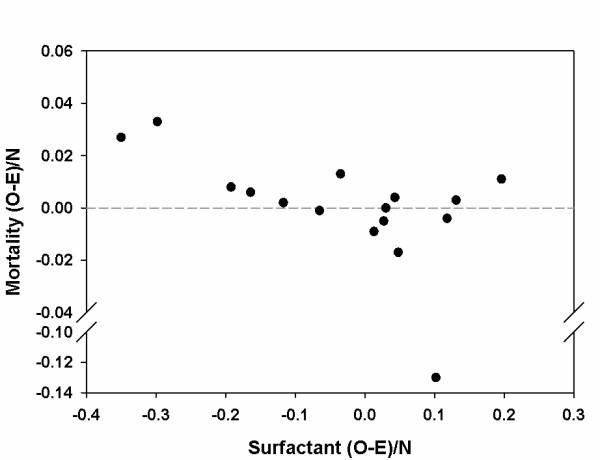
**Association between hospital adjusted rates [(O-E)/N] for surfactant use and mortality**. This scatter plot displays hospital adjusted rates [(O-E)/N] of surfactant use on the x-axis and hospital adjusted rates of mortality [(O-E)/N] on the y-axis (note break in y-axis). Although there was an outlier hospital, the overall trend in the plot is consistent with an association between decreasing mortality and increasing surfactant use (Kendall's tau correlation coefficient of 0.37, p = 0.051).

We compared adjusted hospital rates of surfactant use with adjusted in-hospital mortality rates. The mean bootstrap (O-E)/N values for surfactant use and death and the Haberman *p*-values for each hospital are shown in Table 2. The association between hospital surfactant use (measured by the (O-E)/N statistic for surfactant) and in-hospital NICU mortality (measured by the (O-E)/N statistic for death) is shown in Figure 3. The overall trend in the plot was consistent with an association between decreasing hospital mortality rates and increasing hospital rates of surfactant use (Kendall's tau correlation coefficient of 0.37, implying a probability of concordance of 0.69, p = 0.051). This plot shows one outlier hospital with very low mortality rates. As Kendall's tau is rank-based and robust to outliers, the observed trend was unlikely to be driven by this outlier. We also found a statistically significant correlation between adjusted in-hospital NICU mortality rates and adjusted hospital rates of surfactant use among the population of infants requiring mechanical ventilation (Kendall's tau correlation coefficient of 0.38, also implying a probability of concordance of 0.69, p = 0.047).

**Table 2:** Mean (O-E)/N for hospital surfactant use and death

Hospital	Surfactant use Mean (95% CI) p-value	Death Mean (95% CI) p-value
A	0.102 (0.082,0.122) <0.001*	-0.130 (-0.166,-0.092) <0.001*

B	0.196 (0.079,0.308) 0.010	0.011 (-0.004, 0.026) 0.219

C	0.131 (0.088,0.173) <0.001*	0.003 (-0.006,0.012) 0.573

D	0.118 (0.065,0.172) <0.001*	-0.004 (-0.014,0.006) 0.496

E	0.030 (-0.020,0.082) 0.312	0.00 (-0.012,0.011) 0.999

F	0.048 (0.026,0.071) <0.001*	-0.017 (-0.021,-0.013) <0.001*

G	0.043 (-0.031,0.114) 0.319	0.004 (-0.009,0.017) 0.534

H	0.027 (-0.086,0.137) 0.700	-0.005 (-0.024,0.015) 0.712

I	0.013 (-0.038,0.064) 0.659	-0.009 (-0.018,0.001) 0.129

J	-0.035 (-0.087,0.016) 0.265	0.013 (0.004,0.022) 0.001*

K	-0.065 (-0.113,-0.018) 0.020	-0.001 (-0.010,0.008) 0.845

L	-0.117 (-0.177,-0.057) 0.001*	0.002 (-0.008,0.011) 0.771

M	-0.164 (-0.240,-0.087) <0.001*	0.006 (-0.010,0.022) 0.536

N	-0.192 (-0.269,-0.111) <0.001*	0.008 (-0.006,0.023) 0.472

O	-0.298 (-0.337,-0.258) <0.001*	0.033 (0.023,0.044) <0.001*

P	-0.350 (-0.436,-0.260) <0.001*	0.027 (0.004,0.051) 0.041

*significant with p ≤ 0.05 after Bonferoni correction (p ≤ 0.05/16 = 0.003)

## Discussion

Healthcare providers strive to provide high quality care to their patients. However, in order to determine whether we are actually providing outstanding care, quality measures that are valid, reliable, and easy to collect are needed. We attempted to demonstrate that surfactant use in infants 30 to 34 weeks gestation with RDS may be a useful process measure of hospital quality.

Surfactant use in moderately preterm infants with RDS has the potential to be an ideal quality indicator if it can be accurately measured and can be shown to be a valid measure of quality. The best processes to use as quality indicators are those care practices with strong face validity and evidence linking them to improved patient outcomes. Although there are very few evidence-based therapies in neonatology, the efficacy of surfactant therapy for RDS has been proven in multiple clinical trials and a recent American Academy of Pediatrics report states that surfactant should be given to intubated infants with RDS regardless of exposure to antenatal steroids or gestational age [10]

In an effort to establish the construct validity of surfactant use in infants 30 to 34 weeks' gestation with RDS, we have shown significant variation in rates of surfactant use across hospitals and that hospital surfactant use was associated with variation in two of the most commonly used measures of neonatal quality -- volume of admissions [16-18] and in-hospital mortality [17,19]. These findings are consistent with other examinations of variation in care practices published in the literature. For example, Horbar et al. documented variation in the use of early surfactant among VLBW infants similar to the variation we observed in larger preterm infants with RDS [13]. Additionally, our finding that hospital rates of surfactant use are associated with NICU volume of admissions is consistent with previous reports demonstrating a relationship between NICU volume of admissions and improved mortality [16,17]. While these results are encouraging, limitations in adequate measurement of this quality metric including defining RDS using administrative data, adequately adjusting for severity of illness, and accounting for care received prior to transfer preclude us from establishing the validity of this measure as a marker of neonatal quality of care. Additionally, this study did not attempt to demonstrate the stability of the proposed quality measure over time.

Process measures require accurate identification of the appropriate eligible patient population. Identifying patients with RDS who are eligible for surfactant treatment was particularly challenging using administrative data. The RDS definition used in this study included infants requiring either NCPAP or MV in the first 48 hours of life. We believe this is a reasonable, practical definition for a clinical condition whose definition has varied even across clinical trials of surfactant therapy [4-6]. The definition included both NCPAP and MV in an effort to eliminate the effects of practice variation in ventilatory management across hospitals because the same infant, with the same severity of RDS, may be treated with NCPAP at one center and with MV at another center. However, in doing so, the denominator of eligible patients included some infants who could be successfully managed on NCPAP and some with more mild RDS who might not require surfactant treatment. The fact that there was still significant variation among hospitals in the use of surfactant when we used the ICD-9-CM diagnosis code to identify infants with RDS and when we restricted the population to only those infants requiring mechanical ventilation suggests that true variation exists. However, we acknowledge that there is a potential that systematic differences in RDS severity across hospitals or more liberal use of NCPAP in some centers could explain some of the variation in surfactant rates seen across hospitals. Future efforts to develop process measures in neonatology should focus on care practices with a clear and well-defined eligible patient population.

When the eligible patient population can be defined appropriately, process measures are typically insensitive to differences in case mix and severity of illness [1,26-28]. However, outcome measures of quality such as rates of in-hospital mortality (as used in this study for testing construct validity) require adequate risk adjustment to allow for fair and accurate comparisons across hospitals [27,29-32]. Administrative data is the most accessible comparative database for examining all patients admitted to a hospital and therefore is an important source of data for quality measurement [33]. However, administrative data sets often lack the richness of clinical data sources and they do not typically include physiologic measures of illness severity that can be used for adjustment when making comparisons across hospitals. While it is clear that physiologic measures of illness severity can provide a more nuanced adjustment for differences in case-mix related to illness severity, it is unclear how much these measures add or subtract to adjustments made using demographic and clinical variables as done commonly in other studies of variation in care [16,20]. The variables used to adjust for differences in case-mix when measuring surfactant use and in-hospital mortality in this study included an available subset of the demographic variables used in other studies. This specific combination of variables may not have fully accounted for differences in case-mix across hospitals and for differences in severity of illness; therefore, some of the variation we observed may be attributed to residual differences in case mix that were unaccounted for by our adjustment model. Future efforts to use administrative data to measure neonatal quality will need to first focus on developing valid risk adjustment models. More refined neonatal risk adjustment models are currently under development for use with the PHIS database (personal communication Matt Hall, CHCA).

Many neonatal quality measures are complicated by the impact of patient transfers between hospitals, which is a common occurrence in neonatal and perinatal care [15]. For example, development of valid measures of antenatal steroid use in eligible pregnant women is complicated by the fact that steroids may be given in multiple settings including outpatient clinics and referring hospitals. Measuring surfactant use in infants with RDS is similarly complicated. We do not have information on whether infants received surfactant at an outside hospital prior to transfer. In both our initial analysis and in sensitivity analyses, results were not affected by the day of life when the infant was admitted, making it less likely that receipt of surfactant at an outside hospital influenced whether the infant was truly eligible for surfactant. However, it is possible that variation in surfactant use across hospitals actually reflects differences the care provided before transfer, instead of differences in the quality of care provided by the accepting facility. Future efforts to develop neonatal process measures of quality will likely need to focus on aspects of care that clearly occur at a single location or on developing accurate ways to assess and attribute care provided across multiple locations.

The hospitals that submit data to PHIS are mainly academic children's hospitals. Academic hospitals may vary in their quality of care compared to non-academic hospitals. In addition, we excluded centers with <40 eligible infants in the study period. These smaller NICUs may be less likely to provide high quality care compared to larger NICUs [16-18]. Therefore, additional efforts are needed to generalize these results and to understand how potential quality measures perform in non-academic or smaller NICUs.

## Conclusions

Our study was among the first to use administrative data to examine a process measure reflecting neonatal quality of care. While results were encouraging, challenges defining RDS using administrative data, ensuring adequate risk adjustment, and accounting for care received prior to transfer, did not allow us to draw definitive conclusions about the suitability of rates of surfactant use in infants 30 to 34 weeks' gestation with RDS as a quality indicator. More studies aimed at developing valid and reliable quality measures are needed in order to provide neonatologists with tools to understand and improve the care they provide.

## List of Abbreviations

(RDS): Respiratory Distress Syndrome; (NICU): Neonatal Intensive Care Unit; (OR): Odds Ratio; (CI): Confidence Interval; (VLBW): Very Low Birth Weight; (IRB): Institutional Review Board; (PHIS): Pediatric Health Information Systems; (ICD-9-CM): International Classification of Diseases, 9^th ^Revision, Clinical Modification; (CTC): Clinical Transaction Classification; (APR-DRG): All-Patient Refined Diagnosis-Related Groups; (NCPAP): Nasal Continuous Positive Airway Pressure; (MV): Mechanical Ventilation; (LOS): Length of Stay; (BW): Birth Weight; (ELBW): Extremely Low Birth Weight; (NIH): National Institutes of Health

## Competing interests

The authors declare that they have no competing interests. This work was supported, in part, by NIH grant #K30 HL014134.

## Authors' contributions

HCK was responsible for the study design, data analysis, and interpretation of the data. She also led the drafting of the manuscript. SAL and JHS participated in the design, interpretation of the data, and revising the manuscript for important intellectual content. MP participated in the study design, statistical analysis plan, data analysis, and interpretation of the data. She also revised the manuscript for methodologic content. JPM participated in the study design and manuscript preparation. All authors have read and given final approval of the version of the manuscript to be published.

## Pre-publication history

The pre-publication history for this paper can be accessed here:

http://www.biomedcentral.com/1472-6963/11/22/prepub
